# Overexpression of Lipocalins and Pro-Inflammatory Chemokines and Altered Methylation of PTGS2 and APC2 in Oral Squamous Cell Carcinomas Induced in Rats by 4-Nitroquinoline-1-Oxide

**DOI:** 10.1371/journal.pone.0116285

**Published:** 2015-01-30

**Authors:** Xinjian Peng, Wenping Li, William D. Johnson, Karen E. O. Torres, David L. McCormick

**Affiliations:** 1 Life Sciences Group, IIT Research Institute, Chicago, Illinois, 60616, United States of America; 2 GenUs Biosystems, Northbrook, Illinois, 60062, United States of America; Queen Mary University of London, UNITED KINGDOM

## Abstract

Oral squamous cell carcinomas (OSCC) induced in F344 rats by 4-nitroquinoline-1-oxide (4-NQO) demonstrate considerable phenotypic similarity to human oral cancers. Gene expression studies (microarray and PCR) were coupled with methylation analysis of selected genes to identify molecular markers of carcinogenesis in this model and potential biochemical and molecular targets for oral cancer chemoprevention. Microarray analysis of 11 pairs of OSCC and site-matched phenotypically normal oral tissues from 4-NQO-treated rats identified more than 3500 differentially expressed genes; 1735 genes were up-regulated in rat OSCC versus non-malignant tissues, while 1803 genes were down-regulated. In addition to several genes involved in normal digestion, genes demonstrating the largest fold increases in expression in 4-NQO-induced OSCC include three lipocalins (VEGP1, VEGP2, LCN2) and three chemokines (CCL, CXCL2, CXCL3); both classes are potentially druggable targets for oral cancer chemoprevention and/or therapy. Down-regulated genes in 4-NQO-induced OSCC include numerous keratins and keratin-associated proteins, suggesting that alterations in keratin expression profiles may provide a useful biomarker of oral cancer in F344 rats treated with 4-NQO. Confirming and extending our previous results, PTGS2 (cyclooxygenase-2) and several cyclooxygenase-related genes were significantly up-regulated in 4-NQO-induced oral cancers; up-regulation of PTGS2 was associated with promoter hypomethylation. Rat OSCC also demonstrated increased methylation of the first exon of APC2; the increased methylation was correlated with down-regulation of this tumor suppressor gene. Overexpression of pro-inflammatory chemokines, hypomethylation of PTGS2, and hypermethylation of APC2 may be causally linked to the etiology of oral cancer in this model.

## Introduction

More than 250,000 new cases of oral squamous cell carcinoma (OSCC) are diagnosed each year around the world, and more than 125,000 people die of the disease [1, 2]. In the United States, it is estimated that approximately 28,000 new cases of cancer of the tongue, gum, lip, or mouth will be diagnosed in 2014, and that approximately 8,000 people will die from these malignancies [3]. The most important risk factors for human oral cancer are use of tobacco (including smokeless tobacco) and alcohol [4–6]. It is estimated that consumption of tobacco and alcohol is responsible for approximately 75% of oral cancer cases in the United States, and that tobacco and alcohol may act synergistically to induce oral neoplasia [4, 5]. Exposure to human papillomavirus (HPV) is an emerging factor in oral cancer etiology, and has been identified as a major risk factor in younger individuals as well as in non-smokers and non-drinkers [7, 8].

In spite of continuing efforts to improve surgical and pharmacologic approaches to treat oral cancer, the 5-year survival rate for oral cancer patients has remained between 50% and 60% since the mid-1970’s [9]. On this basis, primary prevention efforts aimed at decreasing exposure to major risk factors for oral cancer, and secondary prevention efforts involving oral cancer chemoprevention are necessary to reduce mortality and the substantial morbidity that is associated with this neoplasm.

The oral cavity provides an attractive site for clinical efforts in cancer prevention, as site accessibility and the existence of grossly identifiable preneoplastic lesions (such as leukoplakia and erythroplakia) facilitate the evaluation of disease progression and chemopreventive drug efficacy. Oral preneoplastic lesions demonstrate a variety of genetic alterations [10, 11], some of which may be critical determinants of lesion progression from preneoplasia to invasive oral cancer.

High quality *in vivo* carcinogenesis models that demonstrate biological congruity with human oral cancer are essential elements of studies to identify molecular targets for oral cancer prevention and to evaluate the efficacy and safety of novel agents and regimens designed to inhibit or retard oral carcinogenesis. An experimental model in which invasive OSCC are induced in the tongue of F344 rats by 4-nitroquinoline 1-oxide (4-NQO) has been used widely in studies of cancer chemoprevention [12–14]. Studies performed in our laboratory [14] demonstrate that invasive oral cancers induced by administration of 4-NQO (20 ppm in the drinking water for ten weeks) develop in four to six months after the first exposure to chemical carcinogen, and demonstrate highly reproducible incidence and latency patterns. Importantly, this model generates invasive malignancies in an anatomic site in which cancers are commonly seen in humans [12, 14]; 4-NQO-induced oral cancers in rats also demonstrate considerable phenotypic similarity to human oral cancers [12].

In the present studies, microarray and PCR approaches were used to identify molecular alterations that are associated with 4-NQO-induced oral carcinogenesis in the F344 rat. The goals of these studies were to identify molecular pathways that could serve as useful targets for oral cancer chemoprevention, and to identify potential biomarkers for carcinogenesis in this site. Additional studies were performed to identify the molecular mechanisms responsible for the differential expression of selected genes in rat oral cancers induced by 4-NQO. We report that oral cancers induced in the rat tongue by 4-NQO demonstrate differential expression of numerous genes, some of which appear to provide suitable targets for pharmacologic interventions directed at oral cancer chemoprevention. Epigenetic changes that include hypomethylation of PTGS2 (COX-2) and hypermethylation of tumor suppressor genes may underlie neoplastic development in this site.

## Materials and Methods

### Animal Welfare Statement

Prior to the initiation of *in vivo* work, the study protocol was reviewed and approved by the IIT Research Institute Animal Care and Use Committee. All elements of the program that involved laboratory animals were performed in full compliance with United States Public Health Service Policy on Humane Care and Use of Laboratory Animals, and in full compliance with the animal welfare guidelines promulgated in the National Research Council *Guide for the Care and Use of Laboratory Animals*.

### *In Vivo* Methods

Six-to-seven week old male F344 rats were obtained from virus-free barrier colonies at Harlan, Frederick, MD. Rats were held in quarantine for approximately one week prior to study start. Cage-side clinical observations were performed daily during the quarantine period to evaluate animal health. Prior to use in the study, each rat received a hand-held physical examination to ensure its suitability for use as a test animal.

At all times during the quarantine and experimental periods, rats were housed on hardwood bedding in polycarbonate shoebox cages in a windowless room that was illuminated for 12 hours per day and maintained within the ranges of 22° ± 1°C and 50% ± 20% relative humidity. Rats were permitted free access to Purina 5001 Laboratory Chow diet (PMI Feeds, Brentwood, MO) and City of Chicago drinking water (provided in water bottles).

All rats received 4-NQO (Sigma-Aldrich, St. Louis, MO) at a concentration of 20 ppm in the drinking water for 10 weeks; after 10 weeks, rats received drinking water without added 4-NQO. After preparation, drinking water containing 4-NQO was stored in the dark at 4°C until used. Bottles containing 4-NQO-supplemented water were wrapped with foil to preclude photodegradation of the carcinogen, and were changed at two- to three-day intervals throughout the study.

Throughout the dosing and observation periods, rats were observed twice daily to evaluate their overall health status and to identify possible toxic effects of the carcinogen. Rats were weighed weekly. Monitoring of body weights is particularly important during latter stages of carcinogenesis studies in the 4-NQO oral cancer model, as body weight loss provides a useful indicator of the clinical progression of induced cancers [14]. The study was terminated at 26 weeks after the first day of carcinogen exposure.

### Necropsy and Histopathology

**Necropsy.** Animals were selected for early euthanasia if hand-held clinical examinations and/or body weight patterns suggested the presence of a large oral cancer; otherwise, all rats were euthanized at 26 weeks after the first exposure to 4-NQO. Animals were humanely euthanized by CO_2_ asphyxiation, and were necropsied immediately to preclude possible artifacts resulting from tissue autolysis. No molecular analyses were performed on tissues from animals that were found dead during the study.

All rats underwent a limited gross necropsy that was focused on the tongue and oral cavity. At necropsy, the tongue from each animal was carefully excised and all gross oral lesions were charted. The tongue was then bisected longitudinally; half of each tongue was fixed in 10% neutral buffered formalin and processed for histopathologic evaluation. The remaining half of each tongue was snap-frozen in liquid N_2_ and stored at -80°C for use in molecular studies.

**Tissue Processing for Microscopic Evaluation.** Formalin-fixed oral tissues collected from 4-NQO-treated rats were processed using standard histologic techniques, cut at 5 µm, stained with hematoxylin and eosin, and classified histopathologically. In addition to qualitative assessment of malignancy, oral cancer invasion scores were determined using a semi-quantitative grading system. The invasiveness of each microscopic oral lesion was scored on a scale of 0 (non-invasive lesion; papilloma or carcinoma *in situ*) to +3 (highly invasive lesion). Malignant lesions with an invasion score of +1 extended through the mucosal epithelial basement membrane, but invaded only into the lamina propria. Malignant lesions with invasion scores of +2 invaded through the lamina propria into the upper muscle layers. Malignant lesions with invasion scores of +3 demonstrated extensive invasion into the underlying muscle.

### Molecular Analyses

**Tissue Processing for Molecular Analyses.** Histologically confirmed OSCC and adjacent phenotypically normal tissue were collected from the half of each tongue that had been designated for molecular analyses. Total DNA/RNA was isolated from paired sets of malignant and normal tissues using the DNeasy Blood & Tissue Kit (Qiagen, Germantown, MD) or RNeasy Mini kit (Qiagen), according to the manufacturer’s instructions. Total RNA was used for microarray and RT-PCR analysis, while total DNA was used for analysis of the methylation status of gene promoters.

**Microarray Analysis.** After RNA isolation, the quality and quantity of individual RNA samples were determined using an Agilent Bioanalyzer. Total RNA isolated from 11 pairs of neoplastic and adjacent normal tongue tissues were individually subjected to microarray analysis using Agilent Rat GE 4x44K v3 arrays (Agilent Technologies, Santa Clara, CA). First and second strand cDNAs were prepared from the total RNA samples; cRNA target was prepared from the DNA template, verified using the Bioanalyzer, fragmented to uniform size, and then hybridized to the microarrays. Slides were washed and scanned using an Agilent G2565 Microarray Scanner. Data were analyzed using Agilent Feature Extraction and GeneSpring GX v7.3.1 software. Microarray data for the 11 sample pairs are available in the National Center for Biotechnology Information Gene Expression Omnibus (GEO), accession GSE51125.

**Quantitative RT-PCR.** RT-PCR analysis was performed as described previously [15]. Two RT reactions for each sample were pooled and diluted with an equal amount of DNase/RNase free water. Real-time PCR was performed with 2 µL diluted RT product in a MyiQ Real-Time PCR Detection System (Bio-Rad, Hercules, CA) using iQ SYBR Green PCR Supermix (Bio-Rad) according to the manufacturer’s instructions. Gene-specific primers were designed using Primer 3 software (http://frodo.wi.mit.edu/cgi-bin/primer3/primer3_www.cgi). Primer sequences are provided in Table A in S1 File (supporting information). 18S ribosomal RNA was used as the reference gene against which qPCR data were normalized. Fold induction was calculated using the formula 2^-(∆∆Ct)^, where ∆∆Ct is ∆Ct_(sample)_ - ∆Ct_(control sample)_, ∆Ct is Ct_(gene)_ - Ct_(18S)_ and Ct is the cycle at which the threshold is crossed. One sample served as a common control for relative fold change calculations.

**Methylation Assay.** Gene methylation in paired samples of tumor and normal tissue were analyzed using the EpiTect Methyl II PCR assay kit (Qiagen). This method is based on detection of remaining input DNA after cleavage with a methylation-sensitive and/or a methylation-dependent restriction enzyme, which digest unmethylated and methylated DNA, respectively.

After digestion, remaining DNA in each individual enzyme reaction was quantified by real-time PCR using primers flanking a promoter region of interest. Relative fractions of methylated and unmethylated DNA were quantitated by comparing the quantity of DNA in each digest to that of a mock digest (no enzymes added) using the ∆Ct method. The primer used in the methylation assay of WIF1 was purchased from Qiagen; other primers were designed based on 600 bp of the proximal promoter sequence 5’-flanking the first exon of the gene of interest. As a first step, the MethPrimer program (http://www.urogene.org/cgi-bin/methprimer/methprimer.cgi [16]) was used to identify a CpG island in the 600 bp promoter sequence; primers were then designed to surround the CpG island. In cases where primers designed using this approach were found to be unsatisfactory for the assay, additional primers were designed based on the 600 bp proximal promoter sequence (which might not cover the CpG island). Detailed information for primer design and CpG island location within the proximal promoter sequence are provided in Table B in S1 File (supporting information). Data are presented both as percent DNA methylation and the ratio of methylation in SCC versus matched normal tissue.

### Statistical analysis

Continuous data were analyzed by Student’s t-test using GraphPad Software (GraphPad, San Diego, CA) and Microsoft Excel. Differences were considered to be significant at p < 0.05.

## Results

### Induction of Oral Cancers in F344 Rats by 4-NQO

**Figure 1:**
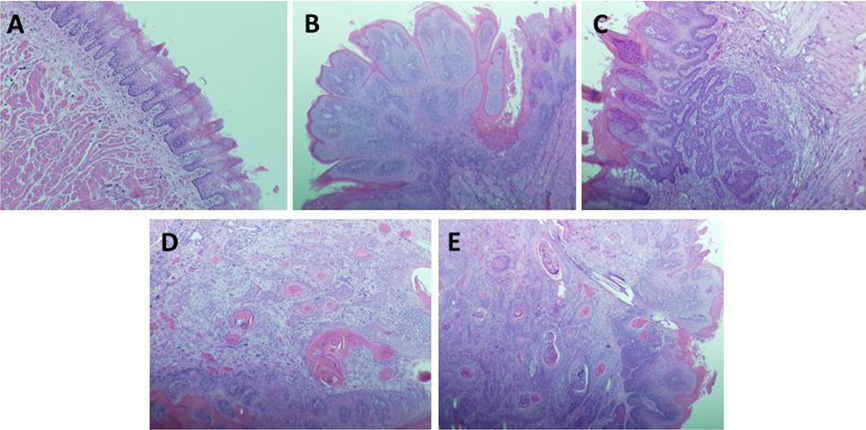
Histology of normal and neoplastic oral (tongue) epithelium in F344 rats treated with 4-NQO. A. Phenotypically normal oral epithelium in F344 rat treated with 4-NQO (H&E, x100). B. Non-invasive squamous cell papilloma (Score 0) induced by 4-NQO. The tumor is confined to the mucosal epithelium of the tongue (H&E, x40). C. Invasive OSCC (Score +1) induced by 4-NQO. The cancer extends through the mucosal epithelial basement membrane into the lamina propria (H&E, x100). D. Invasive OSCC (Score +2) induced by 4-NQO. The cancer extends into the upper muscle layers of the tongue (H&E, x100). E. Invasive OSCC (Score +3) induced by 4-NQO. The cancer demonstrates extensive invasion into the underlying muscle layers of the tongue (H&E, x40).

Two separate oral cancer induction experiments (Table 1) were performed to generate tissues for molecular analysis. In these studies, drinking water administration of 4-NQO (20 ppm for 10 weeks) to F344 rats induced a range of premalignant and invasive malignant lesions (hyperplasia, squamous cell papilloma, and OSCC) in the tongue. Photomicrographs demonstrating the normal histology of the tongue (Panel A), a typical squamous cell papilloma (Panel B), and squamous cell carcinomas with invasion scores of +1 (Panel C), +2 (Panel D), and +3 (Panel E) are provided in Fig. 1.

**Table 1:** Oral cancer incidence and invasion score in male F344 rats treated with 4-NQO.

**Experiment Number**	**Number of Rats**	**Number (%) of Animals with Lesion / Number of Animals at Risk**				
		**Normal**	**Squamous Epithelial Hyperplasia**	**Squamous Cell Papilloma**	**Squamous Cell Carcinoma**	**Carcinoma Invasion Score**
1	30	0/30 (0)	3/30 (10)	2/30 (7)	25/30 (83)	
					1/30 (3)	+1 Invasion
					4/30 (13)	+2 Invasion
					20/30 (67)	+3 Invasion
2	28	0/28 (0)	3/28 (11)	4/28 (14)	21/28 (75)	
					2/28 (7)	+1 Invasion
					0/28 (0)	+2 Invasion
					19/28 (68)	+3 Invasion

The induction of invasive OSCC by 4-NQO was highly reproducible: 83% and 75% of rats in the two experiments demonstrated invasive oral cancers at six months after the start of carcinogen administration (Table 1), and 67% and 68% of 4-NQO-treated rats in the two studies demonstrated highly invasive oral cancers (+3 invasion score). Oral cancer incidences and invasion scores seen in these two experiments are consistent with the results of previous studies performed in our laboratory using this model [14].

### Microarray Analyses of Oral Cancers induced in F344 Rats by 4-NQO

Microarray analyses were performed on 11 matched tissue pairs from Experiment 1 to compare patterns of gene expression at the mRNA level in OSCC and adjacent phenotypically normal oral tissues. Using a fold-change cutoff value of 2.0 for differences in gene expression between normal and neoplastic oral tissues and a significance cutoff value of p < 0.05, 3538 genes were differentially expressed in OSCC versus adjacent, phenotypically normal oral tissues (S1 Fig., supporting information). Of these differentially expressed genes, 1735 genes were significantly up-regulated and 1803 genes were significantly down-regulated in OSCC.

Annotated genes demonstrating the largest fold changes in expression in OSCC induced by 4-NQO are listed in Table 2 (up-regulated genes) and Table 3 (down-regulated genes). Genes demonstrating the greatest up-regulation in OSCC were commonly characterized by very low expression in phenotypically normal oral tissues, and 25- to 75-fold overexpression in cancers (Table 2). Up-regulated genes in OSCC induced by 4-NQO include several lipocalins (*e.g.,* von Ebners gland proteins 1 and 2 (VEGP1 and VEGP2) and lipocalin2 (LCN2; neutrophil gelatinase-associated lipocalin (NGAL)). LCN2 has also been demonstrated to be up-regulated in human OSCC [17] and in pancreatic, colon, skin, and breast cancers in humans [18–21]; knockdown of LCN2 inhibits the growth and invasiveness of prostate cancer cells [22]. On the basis of its overexpression in human cancers and apparent pro-inflammatory activity and stimulatory effects on tumor growth, LCN2 has been proposed as a therapeutic target for cancer [22, 23].

**Table 2:** Genes demonstrating the greatest up-regulation in OSCC versus adjacent normal oral tissues.

**Gene Symbol**	**Primary Accession**	**Gene Name**	**Normal (Mean ± SD)**	**OSCC (Mean ± SD)**	**Expression Ratio (OSCC/Normal)**	**p value**
Vegp2	NM_053574	von Ebners gland protein 2	0.03 ± 0.05	1.95 ± 0.19	76.17	4.04 × 10^-7^
Vegp1	NM_022945	von Ebners gland protein 1	0.03 ± 0.05	1.94 ± 0.09	74.67	4.57 × 10^-7^
Bpifb1	NM_001077680	BPI fold containing family B, member 1	0.03 ± 0.09	1.79 ± 0.58	67.59	3.59 × 10^-6^
Lipf	NM_017341	lipase, gastric	0.03 ± 0.08	1.92 ± 0.19	62.20	2.34 × 10^-6^
Mmp12	NM_053963	matrix metallopeptidase 12	0.04 ± 0.15	1.83 ± 0.30	51.66	1.55 × 10^-5^
Bpifa1	NM_172031	BPI fold containing family A, member 1	0.04 ± 0.14	1.85 ± 0.26	48.65	1.32 × 10^-5^
LOC171161	NM_133622	common salivary protein 1	0.04 ± 0.16	1.76 ± 0.54	48.63	1.72 × 10^-5^
Mzb1	NM_001024240	marginal zone B and B1 cell-specific protein	0.04 ± 0.08	1.92 ± 0.25	47.93	1.15 × 10^-6^
Hp	NM_012582	haptoglobin	0.04 ± 0.06	1.92 ± 0.26	45.00	3.07 × 10^-7^
Cxcl2	NM_053647	chemokine (C-X-C motif) ligand 2	0.04 ± 0.18	1.77 ± 0.52	44.97	2.58 × 10^-5^
Lcn2	NM_130741	lipocalin 2	0.05 ± 0.08	1.89 ± 0.23	41.88	1.38 × 10^-6^
Ccl3	NM_013025	chemokine (C-C motif) ligand 3	0.05 ± 0.21	1.81 ± 0.29	36.03	3.81 × 10^-5^
Prb1	NM_172065	proline-rich protein BstNI subfamily 1	0.05 ± 0.40	1.61 ± 0.67	31.84	1.92 × 10^-4^
Amy1a	NM_001010970	amylase, alpha 1A (salivary)	0.06 ± 0.38	1.60 ± 0.74	28.61	1.76 × 10^-4^
Postn	NM_001108550	periostin, osteoblast specific factor	0.06 ± 0.22	1.75 ± 0.42	27.64	4.01 × 10^-5^
Spp1	NM_012881	secreted phosphoprotein 1	0.07 ± 0.18	1.79 ± 0.31	26.72	1.75 × 10^-5^
S100a9	NM_053587	S100 calcium binding protein A9	0.07 ± 0.24	1.74 ± 0.48	26.08	5.04 × 10^-5^
Cxcl3	D87927	chemokine (C-X-C motif) ligand 3	0.05 ± 0.54	1.41 ± 1.25	26.06	4.31 × 10^-4^
Klk6	NM_019175	kallikrein related-peptidase 6	0.07 ± 0.18	1.75 ± 0.52	24.73	1.50 × 10^-5^
Bhlha15	NM_012863	basic helix-loop-helix family, member a15	0.07 ± 0.28	1.78 ± 0.50	24.48	7.50 × 10^-5^

Gene expression data are derived from raw intensity values normalized to the 75th percentile of each array, followed by calculation of mean expression in paired OSCC and phenotypically normal oral tissues; n = 11 tissue pairs). P values were calculated by paired Student’s t-test using GeneSpring software

**Table 3:** Genes demonstrating the greatest down-regulation in OSCC versus adjacent normal oral tissues.

**Gene Symbol**	**Primary Accession**	**Gene Name**	**Normal (Mean ± SD)**	**OSCC (Mean ± SD)**	**Expression Ratio (OSCC/Normal)**	**p value**
Krtap13-2	NM_001109325	keratin associated protein 13-2	1.60 ± 1.03	0.04 ± 0.21	0.02	3.40 × 10^-5^
Krt34	NM_001008758	keratin 34	1.53 ± 1.32	0.05 ± 0.19	0.03	2.47 × 10^-5^
Sbk2	NM_001127539	SH3-binding domain kinase family, mbr 2	1.80 ± 0.31	0.06 ± 0.20	0.03	3.29 × 10^-5^
Krtap4-3	XM_001081433	keratin associated protein 4-3	1.72 ± 0.70	0.06 ± 0.17	0.03	1.44 × 10^-5^
Krtap4-7	XM_002724553	keratin associated protein 4-7	1.75 ± 0.63	0.06 ± 0.14	0.03	5.70 × 10^-6^
Krt33b	NM_001008819	keratin 33B	1.51 ± 1.35	0.05 ± 0.20	0.03	2.62 × 10^-5^
Krt35	NM_001008820	keratin 35	1.72 ± 0.64	0.07 ± 0.19	0.04	2.09 × 10^-5^
Isl1	NM_017339	ISL LIM homeobox 1	1.87 ± 0.30	0.08 ± 0.10	0.04	5.24 × 10^-7^
Krtap1-3	ENSRNOT00000037980	keratin associated protein 1-3	1.66 ± 0.80	0.08 ± 0.22	0.05	2.74 × 10^-5^
Tlx1	NM_001109166	T-cell leukemia, homeobox 1	1.89 ± 0.19	0.09 ± 0.06	0.05	1.90 × 10^-8^
Krtap3-3l1	XM_003750924	keratin associated protein 3-3-like 1	1.63 ± 0.78	0.09 ± 0.27	0.05	5.91 × 10^-5^
Krtap2-4l	XM_003752376	keratin associated protein 2-4-like	1.66 ± 0.76	0.09 ± 0.23	0.05	2.89 × 10^-5^
Myh4	NM_019325	myosin, heavy chain 4, skeletal muscle	1.64 ± 0.51	0.10 ± 0.45	0.06	3.20 × 10^-4^
Krt33a	NM_001008757	keratin 33A	1.45 ± 1.35	0.09 ± 0.32	0.06	1.41 × 10^-4^
Krtap7-1	NM_001145002	keratin associated protein 7-1	1.66 ± 0.74	0.11 ± 0.19	0.07	9.48 × 10^-6^
Aox4	NM_001008523	aldehyde oxidase 4	1.78 ± 0.23	0.12 ± 0.24	0.07	3.60 × 10^-5^
Tmem179	NM_001126280	transmembrane protein 179	1.76 ± 0.29	0.12 ± 0.19	0.07	1.11 × 10^-5^
Dsg4	NM_199490	desmoglein 4	1.72 ± 0.44	0.12 ± 0.23	0.07	2.33 × 10^-5^
Krtap14l	ENSRNOT00000064058	keratin associated protein 14 like	1.42 ± 1.13	0.11 ± 0.47	0.08	5.19 × 10^-4^
Asb9	NM_001191913	ankyrin repeat and SOCS box-containing 9	1.83 ± 0.24	0.14 ± 0.10	0.08	4.94 × 10^-8^

Several chemokine ligands (chemokine (C-C motif) ligand 3 (CCL3), chemokine (C-X-C motif) ligand 2 (CXCL2), and chemokine (C-X-C motif) ligand 3 (CXCL3)) also demonstrated >25-fold overexpression in 4-NQO-induced oral cancers. In addition to these abundantly over-expressed chemokine ligands, numerous other chemokines and chemokine receptors were also significantly up-regulated in OSCC versus adjacent normal oral tissues (Table 4). The only exception to this pattern of differential expression was the apparent down-regulation of CCL11 in OSCC (Table 4). Increased production of chemokine ligands may contribute to the development of human OSCC [24, 25] and other malignancies [26, 27] through proinflammatory and other immunomodulatory mechanisms. Chemokines have also been proposed to be druggable targets for cancer therapy [28, 29].

**Table 4:** Comparative expression of chemokine ligands and receptors in OSCC and adjacent normal oral tissues.

**Gene Symbol**	**Primary Accession**	**Gene Name**	**Normal (Mean ± SD)**	**OSCC (Mean ± SD)**	**Expression Ratio (OSCC/Normal)**	**p value**
Ccl9	NM_001012357	chemokine (C-C motif) ligand 9	0.11 ± 0.43	1.55 ± 0.69	14.33	3.21 × 10^-4^
Ccl12	NM_001105822	chemokine (C-C motif) ligand 12	0.19 ± 0.29	1.63 ± 0.48	8.63	5.74 × 10^-5^
Ccl4	NM_053858	chemokine (C-C motif) ligand 4	0.23 ± 0.53	1.44 ± 0.64	6.19	1.09 × 10^-3^
Ccl2	NM_031530	chemokine (C-C motif) ligand 2	0.24 ± 0.40	1.43 ± 0.71	6.03	3.73 × 10^-4^
Ccl6	NM_001004202	chemokine (C-C motif) ligand 6	0.29 ± 0.16	1.66 ± 0.25	5.787	2.66 × 10^-7^
Ccl7	NM_001007612	chemokine (C-C motif) ligand 7	0.31 ± 0.49	1.37 ± 0.62	4.47	1.20 × 10^-3^
Ccl20	NM_019233	chemokine (C-C motif) ligand 20	0.57 ± 0.38	1.28 ± 0.43	2.26	1.35 × 10^-3^
Ccl17	NM_057151	chemokine (C-C motif) ligand 17	0.52 ± 0.55	1.17 ± 0.68	2.25	1.73 × 10^-2^
Ccl11	NM_019205	chemokine (C-C motif) ligand 11	1.51 ± 0.41	0.37 ± 0.27	0.24	1.64 × 10^-5^
Cxcl17	NM_001107491	chemokine (C-X-C motif) ligand 17	0.13 ± 0.13	1.59 ± 0.61	12.29	6.53 × 10^-4^
Cxcl13	NM_001017496	chemokine (C-X-C motif) ligand 13	0.12 ± 0.54	1.50 ± 0.78	12.24	6.54 × 10^-4^
Cxcl1	NM_030845	chemokine (C-X-C motif) ligand 1	0.14 ± 0.48	1.50 ± 0.65	10.64	5.38 × 10^-4^
Cxcl16	NM_001017478	chemokine (C-X-C motif) ligand 16	0.58 ± 0.28	1.31 ± 0.40	2.28	1.20 × 10^-4^
Ccr1	NM_020542	chemokine (C-C motif) receptor 1	0.24 ± 0.37	1.56 ± 0.46	6.49	1.88 × 10^-4^
Ccr2	NM_021866	chemokine (C-C motif) receptor 2	0.34 ± 0.32	1.52 ± 0.35	4.46	7.15 × 10^-5^
Ccr5	NM_053960	chemokine (C-C motif) receptor 5	0.39 ± 0.27	1.50 ± 0.33	3.83	1.56 × 10^-5^
Ccr7	NM_199489	chemokine (C-C motif) receptor 7	0.43 ± 0.31	1.42 ± 0.44	3.28	6.85 × 10^-5^
Ccrl2	NM_001108191	chemokine (C-C motif) receptor-like 2	0.52 ± 0.34	1.33 ± 0.41	2.55	3.44 × 10^-4^
Cxcr2	NM_017183	chemokine (C-X-C motif) receptor 2	0.21 ± 0.39	1.61 ± 0.35	7.71	2.31 × 10^-4^
Cxcr6	NM_001102587	chemokine (C-X-C motif) receptor 6	0.23 ± 0.29	1.62 ± 1.62	7.16	4.75 × 10^-5^
Cx3cr1	NM_133534	chemokine (C-X3-C motif) receptor 1	0.30 ± 0.33	1.46 ± 1.46	4.92	9.86 × 10^-5^
Cxcr4	NM_022205	chemokine (C-X-C motif) receptor 4	0.44 ± 0.31	1.44 ± 0.34	3.29	7.04 × 10^-5^

Other highly up-regulated genes in OSCC include digestive enzymes (gastric lipase and α-amylase) and the serine protease, kallikrein related peptidase 6 (KLK6). In consideration of their central roles in normal digestion, neither gastric lipase nor α-amylase presents a suitable target for pharmacologic intervention. By contrast, as a result of its upregulation in human colon cancer [30], gastric cancer [31], ovarian cancer [32] and melanoma [33], KLK6 has been identified as a potential target for pharmacologic intervention, as well as a prognostic indicator for disease progression and survival [34, 35].

Of the 20 genes demonstrating the greatest down-regulation in OSCC induced by 4-NQO, twelve were keratins or keratin-associated proteins (Table 3). These data provide clear evidence of altered patterns of epithelial differentiation within OSCC. In addition to the twelve very highly down-regulated genes for keratins and keratin-associated proteins, genes for more than twenty additional keratins or keratin-associated proteins demonstrated more than two-fold differential expression in NQO-induced OSCC (Table 5). Alterations in keratin profiles have been reported in OSCC in several species, including humans [36–38], and as such have significant potential as biomarkers of oral neoplasia.

**Table 5:** Differentially expressed keratins and keratin-associated proteins in OSCC versus adjacent normal oral tissues.

**Gene Symbol**	**Primary Accession**	**Gene Name**	**Normal (Mean ± SD)**	**OSCC (Mean ± SD)**	**Expression Ratio (OSCC/Normal)**	**p value**
Krt18	NM_053976	keratin 18	0.09 ± 0.49	1.64 ± 0.55	19.10	3.67 × 10^-4^
Krt8	NM_199370	keratin 8	0.10 ± 0.17	1.79 ± 0.33	18.72	8.38 × 10^-6^
Krt7	XM_003750407	keratin 7	0.10 ± 0.28	1.66 ± 0.52	16.70	7.49 × 10^-5^
Krt20	NM_173128	keratin 20	0.25 ± 0.25	1.63 ± 0.35	6.55	1.48 × 10^-5^
Krt16	NM_001008752	keratin 16	0.23 ± 0.43	1.11 ± 2.96	4.86	1.22 × 10^-2^
Krt80	NM_001008815	keratin 80	0.34 ± 0.36	1.48 ± 0.46	4.33	1.71 × 10^-4^
Krt12	NM_001008761	keratin 12	0.33 ± 0.37	1.37 ± 0.83	4.12	3.63 × 10^-4^
Krt1	NM_001008802	keratin 1	0.35 ± 0.60	1.14 ± 1.44	3.23	1.88 × 10^-2^
Krt10	NM_001008804	keratin 10	0.40 ± 0.47	1.27 ± 0.89	3.15	3.35 × 10^-3^
Krt19	NM_199498	keratin 19	0.50 ± 0.70	1.19 ± 0.52	2.37	2.41 × 10^-2^
Krt24	NM_001004131	keratin 24	1.25 ± 0.57	0.39 ± 1.06	0.31	2.67 × 10^-2^
Krt31	NM_001008817	keratin 31	1.15 ± 1.09	0.29 ± 1.06	0.25	2.38 × 10^-2^
Krt36	NM_001008759	keratin 36	1.35 ± 0.90	0.27 ± 0.56	0.20	2.20 × 10^-3^
Krt83	NM_001101675	keratin 83	1.20 ± 1.58	0.21 ± 0.46	0.18	2.60 × 10^-3^
Krt15	NM_001004022	keratin 15	1.68 ± 0.17	0.29 ± 0.15	0.17	1.62 × 10^-7^
Krt85	NM_001008811	keratin 85	1.16 ± 1.94	0.18 ± 0.50	0.16	3.93 × 10^-3^
Krtap1-1	ENSRNOT00000041345	keratin associated protein 1-1	1.59 ± 0.49	0.24 ± 0.28	0.15	3.01 × 10^-5^
Krtap3-1	ENSRNOT00000016752	keratin associated protein 3-1	1.36 ± 1.23	0.19 ± 0.39	0.14	4.93 × 10^-4^
Krtap8-1	ENSRNOT00000064583	keratin associated protein 8-1	1.53 ± 0.58	0.21 ± 0.38	0.14	2.13 × 10^-4^

It should be noted that some variability in patterns of gene expression was seen in both normal oral tissues and in OSCC from several animals (*e.g*., animal numbers 21, 22 and 24; S1 Fig.). A possible reason for the observed heterogeneity in gene expression profiles in phenotypically normal tissues is the presence of cells that are grossly and microscopically normal, yet demonstrate genetic damage or dysregulated gene expression in response to 4-NQO; these cells might be expected to progress to neoplasia with additional time. The observed heterogeneity in gene expression profiles in OSCC induced by 4-NQO could also reflect the induction of neoplasia through different molecular pathways in different animals.

### Microarray and qRT-PCR Analyses of COX-Related Genes in Oral Cancers

We have previously reported that COX-2 is significantly overexpressed in OSCC induced in rats by 4-NQO, and that oral carcinogenesis in this model can be inhibited by both selective COX-2 inhibitors (*e.g.,* celecoxib) and nonselective COX inhibitors (*e.g*., piroxicam and naproxen) [14]. Consistent with our previous results, KEGG pathway and gene ontology analyses of microarray data from tissues generated in Experiment 1 identified arachidonic acid metabolism as a significantly altered pathway in rat OSCC. To further investigate the expression of COX-1 (PTGS1), COX-2 (PTGS2), and COX-related genes in rat oral cancers, a list of COX-related genes was generated using Ingenuity software (http://www.ingenuity.com); integration of this gene list with microarray data from Experiment 1 identified a series of COX-related genes that are up-regulated in rat oral cancers induced by 4-NQO (Table 6). From this list, 5 genes (PTGS2, MMP9, NOS2, VEGFα, and IL1β) were selected for confirmation by qRT-PCR using a different set of tissue samples (8 tissue pairs from Experiment 2). qRT-PCR analysis of these 8 tissue pairs confirmed the upregulation of all selected genes in OSCC (S2 Fig., supporting information). In addition to the five COX-related genes, upregulation of three additional matrix metalloproteases (MMP10, MMP12, and MMP13; data not shown) and TGFα (S2 Fig.) in OSCC were also confirmed by qRT-PCR.

**Table 6:** Overexpression of COX (PTGS1 and PTGS2) and COX-related genes in OSCC versus adjacent normal oral tissues.

**Gene Symbol**	**Primary Accession**	**Gene Name**	**Normal (Mean ± SD)**	**OSCC (Mean ± SD)**	**Expression Ratio (OSCC/Normal)**	**P value**
PTGS1	NM_017043	Prostaglandin-endoperoxide synthase 1	0.54 ± 0.31	1.34 ± 0.39	2.47	1.63 × 10^-4^
PTGS2	NM_017232	Prostaglandin-endoperoxide synthase 2	0.35 ± 0.43	1.65 ± 0.43	12.28	3.86 × 10^-4^
**Genes Regulated by PGST2**						
MMP9	XM_0301055	Matrix metallopeptidase 9	0.11 ± 0.52	1.37 ± 1.26	12.96	7.43 × 10^-4^
BCL2α	NM_133416	BCL2-related protein A1	0.25 ± 0.39	1.49 ± 0.56	6.00	2.59 × 10^-4^
NOS2	NM_012611	Nitric oxide synthase 2, inducible	0.32 ± 0.44	1.44 ± 0.50	4.46	6.08 × 10^-4^
TNF	NM_012675	Tumor necrosis factor	0.50 ± 0.46	1.50 ± 0.46	4.32	5.48 × 10^-4^
PTGER4	NM_032076	Prostaglandin E receptor 4 (subtype EP4)	0.58 ± 0.19	1.42 ± 0.19	2.54	2.41 × 10^-6^
VEGFα	XM_001110334	Vascular endothelial growth factor A	0.64 ± 0.18	1.36 ± 0.18	2.20	8.00 × 10^-7^
**Genes Regulating PGST2**						
IL1β	NM_031512	Interleukin 1 β	0.09 ± 0.34	1.67 ± 0.51	17.71	1.42 × 10^-6^
IL1α	NM_017019	Interleukin 1 α	0.15 ± 0.28	1.68 ± 0.53	11.25	5.16 × 10^-5^
TNF	NM_012675	Tumor necrosis factor	0.50 ± 0.46	1.50 ± 0.46	4.32	5.48 × 10^-4^

### Methylation Analysis of PTGS2 proximal promoter

As discussed above (and confirming our previous findings), microarray analysis of PTGS2 expression in eleven tissue pairs from Experiment 1 and qRT-PCR analysis of PTGS2 expression in eight tissue pairs from Experiment 2 demonstrated that PTGS2 expression is upregulated by more than 10-fold in OSCC when compared to adjacent phenotypically normal tissues. To determine whether the upregulation of PTGS2 in oral cancers is the result of its hypomethylation, studies were performed to characterize the methylation status of the PTGS2 proximal promoter in the eight pairs of tumor and normal oral tissue samples from Experiment 2.

Initially, the 600 bp 5’ flanking sequence of the transcription start site of PTGS2 was analyzed using MethPrimer to identify potential CpG islands for analysis. One CpG island (from 345 bp to 494 bp of the sequence) was identified, and three pairs of primers (P1, P2, and P3) were designed to cover this CpG island (S3 Fig., supporting information). In addition, a fourth pair of primers (P4) was designed to analyze the methylation status of non-CpG regions of the promoter. Assays using primers P1, P2, and P3 failed to generate meaningful data for methylation status of the CpG island region of the PTGS2 proximal promoter; however, methylation assays with primer P4 demonstrated that this non-CpG region was substantially hypomethylated in OSCC versus adjacent normal tissue (Table 7). DNA from normal oral tissues demonstrated a mean of 18.9% methylation in the PTGS2 promoter, as compared to 2.16% methylated DNA in this region in OSCC (p < 0.05). If hypomethylation is defined as a methylation ratio of < 0.5 in OSCC versus normal oral tissue, 5 of 8 DNA samples from OSCC were hypomethylated in the proximal promoter region of PTGS2.

**Table 7:** Methylation status of the proximal promoter of PTGS2 in OSCC versus adjacent normal oral tissues.

**Sample I.D.**	**% Methylation (OSCC)**	**% Methylation (Normal Tissue)**	**Methylation Ratio (OSCC/Normal)**
A	1.11	2.45	0.45
B	1.24	29.9	0.04
C	3.29	5.83	0.56
D	1.14	25.7	0.04
E	1.03	1.43	0.72
F	2.10	63.3	0.03
G	4.23	21.2	0.20
H	3.16	1.54	2.05
**Mean ± S.E.M.**	2.16* ± 0.39	**18.9 ± 7.55**	

*p < 0.05 in comparison to corresponding normal tissue

### Methylation Analysis of Proximal Promoter Regions of Tumor Suppressor Genes

Because hypomethylation of its proximal promoter is linked to the observed upregulation of PTGS2 in OSCC, we hypothesized that downregulation of tumor suppressor gene expression in OSCC may be related to hypermethylation of their proximal promoters. To test this hypothesis, methylation profiles were generated for five potential tumor suppressor genes that were found to be downregulated in OSCC induced by 4-NQO. Putative tumor suppressor genes were selected for analysis on the basis of microarray data from Experiment 1 and evaluation of literature data; the strategy described above for PTGS2 was used to determine the methylation profiles of the promoter regions of these genes.

Genes selected for analysis were cyclin A1 (CCNA1); Hras-like suppressor (HRASLS); DNA damage-inducible transcript 4-like (DDIT4L), Wnt inhibitory factor 1 (WIF1) and adenomatosis polyposis coli 2 (APC2). All selected genes consistently demonstrated a minimum of 2-fold downregulation in the 11 sample pairs analyzed in Experiment 1 (p < 0.001; Table C in S1 File (supporting information)).

Methylation of CpG islands in the proximal promoter regions of CCNA1, HRASLS, DDIT4L and WIF1 was relatively low (mean methylation of <10% for all genes), and substantial interindividual variability was seen in the methylation of each gene. Increased methylation of the promoter region of HRASLA was identified in OSCC in four of eight tissue pairs (Table 8); however, neither this difference nor the small differences seen in promoter methylation of CCNA1, DDIT4L, or WIF1 in OSCC was significantly different from normal tissues at the 5% level of confidence.

**Table 8:** Methylation profiles of the proximal promoters (600 bp 5’flanking sequence) of CCNA1, HRASLS, DDIT4L, WIF1 and APC2 in OSCC versus adjacent normal oral tissues.

**Sample No.**	**CCNA1**			**HRASLS**			**DDIT4L**			**WIF1**			**APC2**		
	**M% (N)**	**M% (OSCC)**	**Ratio (OSCC/N)**	**M% (N)**	**M% (OSCC)**	**Ratio (OSCC/N)**	**M% (N)**	**M% (OSCC)**	**Ratio (OSCC/N)**	**M% (N)**	**M% (OSCC)**	**Ratio (OSCC/N)**	**M% (N)**	**M% (OSCC)**	**Ratio (OSCC/N)**
1	1.39	0.54	0.39	0.89	1.91	2.15	4.62	4.19	0.91	1.93	1.79	0.93	90.07	92.93	1.03
5	0.53	1.18	2.23	1.28	0.85	0.66	4.47	3.66	0.82	1.37	1.08	0.79	88.79	95.70	1.08
7	2.20	1.20	0.55	0.71	2.38	3.35	9.31	13.68	1.47	0.98	3.20	3.27	72.97	95.15	1.30
8	0.99	1.10	1.11	1.11	0.31	0.28	5.18	2.15	0.42	1.39	1.02	0.73	90.27	94.97	1.05
9	1.57	1.03	0.66	1.55	0.43	0.28	4.22	5.55	1.32	1.17	1.20	1.03	90.36	94.51	1.05
14	2.68	2.59	0.97	0.97	1.17	1.21	7.08	7.69	1.09	1.67	1.23	0.74	85.88	91.55	1.07
23	1.84	1.68	0.91	0.72	13.58	18.86	8.09	5.91	0.73	1.64	2.00	1.22	88.65	97.10	1.10
30	2.07	1.32	0.64	0.64	0.56	0.88	4.96	3.11	0.63	1.83	0.99	0.54	92.49	96.30	1.04
Mean	1.66	1.33	0.93	0.98	2.65	3.46	5.99	5.74	0.92	1.50	1.56	1.15	87.44	94.78**	1.09
SEM	0.24	0.21	0.20	0.11	1.58	2.23	0.68	1.29	0.12	0.12	0.27	0.31	2.171	0.64	0.03

M%, Methylation % N, normal OSCC, Oral Squamous Cell Carcinoma

**p<0.01 in comparison to corresponding normal control

No CpG islands were identified in the proximal promoter region of APC2; however, its first exon was highly methylated (87.4 ± 2.17%) in normal oral tissues. In OSCC, the first exon of APC2 was hypermethylated, demonstrating a mean methylation level of 94.8 ± 0.64% (p < 0.01 versus phenotypically normal oral tissue; Table 7). Although these data are clearly suggestive, the role of increased methylation in the first exon of APC2 as a mediator of the decreased expression of this gene in OSCC remains to be definitively demonstrated.

## Discussion

The overall goals of the present studies were to (a) identify potential molecular targets for oral cancer chemoprevention; (b) identify molecular signatures that may serve as biomarkers of oral carcinogenesis; and (c) explore the role of promoter methylation as a possible mechanism for the differential expression of selected genes in oral cancers.

Drinking water administration of 4-NQO provides a reproducible method to induce invasive OSCC in rats without the induction of systemic toxicity; the sequential changes in oral epithelial morphology in rats treated with 4-NQO resemble those reported during progression of OSCC in humans [12]. Although most oral cancer chemoprevention studies using the 4-NQO/rat model have been performed in F344 rats, the induction of OSCC in rats by 4-NQO has also been studied in a variety of other rat strains. In a study involving seven strains of rats (Dark-Agouti, Long-Evans, Sprague-Dawley, ACI/Ms, F344, Donryu, and Wistar/Furth), Kitano and colleagues reported substantial inter-strain differences in OSCC responses to 4-NQO; Dark-Agouti rats were the most sensitive to the induction of oral carcinogenesis by 4-NQO, while Wistar/Furth rats were the least sensitive [39].

Genes that demonstrate the greatest overexpression in OSCC induced by 4-NQO include lipocalins and chemokine ligands. Three lipocalins (VEGP1, VEGP2, and LCN2) were upregulated by > 25-fold in OSCC induced in rats by 4-NQO; LCN2 is also upregulated in human OSCC [17] and in several other human malignancies [18–21]. These data extend our previous findings with COX-2 by providing additional evidence of the molecular similarity between oral cancers induced in rats by 4-NQO and human oral cancers. Furthermore, when considered with the proproliferative, antiapoptotic, and proinflammatory effects of LCN2 [40], these data suggest that LCN2 and/or other lipocalins may provide useful targets for the design of novel agents for oral cancer chemoprevention. Of particular interest in this regard is the finding that LCN2 is upregulated in HPV-positive keratinocytes and cutaneous SCC [20]. Taken together, the observed upregulation of LCN2 in oral cancers and its apparent upregulation by HPV infection suggest that this gene may provide a mechanistically relevant target for the prevention of oral neoplasia induced by HPV. In this regard, the value of chemopreventive interventions targeting LCN2 may increase over time, as the fraction of oral cancers that are linked to HPV infection continues to increase [7, 41]. It must be noted, however, that although knockdown of LCN2 has been demonstrated to inhibit the proliferation of tumor cells *in vitro* [22], the feasibility of specific pharmacologic modulation of LCN2 expression *in vivo* with either small molecule therapeutics or biotherapeutic agents has not been demonstrated.

The chemokine ligands, CCL3, CXCL2, and CXCL3, were each overexpressed by > 25-fold in NQO-induced oral cancers. CCL3 is also overexpressed in human oral cancers [24], providing additional evidence of the molecular relevance of the 4-NQO rat oral cancer model to human oral malignancy. It has been proposed that chemokine ligands may support neoplastic development in the oral cavity through proinflammatory or immunomodulatory mechanisms [24, 25]; this hypothesis is supported by our studies demonstrating potent inhibition of oral carcinogenesis by non-steroidal anti-inflammatory agents [14]. Although its role in oral carcinogenesis has not been studied, CXCL2 has been reported be involved in destruction of bone by OSCC [42].

Recent evidence suggests that CCL2 and other chemokine ligands may present both molecularly relevant and pharmacologically feasible targets for the design of small molecule drugs or biotherapeutics. CCL2 was found to be upregulated by >6 fold in 4-NQO induced rat OSCC in the present studies, and has been proposed as a potential biomarker for the diagnosis of OSCC in humans [43]. CCL2 has also been reported to be an important mediator of the enhancement of OSCC growth by cancer-associated fibroblasts [25]. CCL2 also appears to be a druggable molecular target, as data from a Phase 1 clinical trial of a monoclonal antibody targeting CCL2 were recently reported [29].

The serine protease, KLK6, is also overexpressed by more than 25-fold in rat oral cancers induced by 4-NQO. Although differential expression of KLK6 in human oral cancers has not been reported, KLK6 is significantly upregulated in cancers of the colon and several other sites in humans [30–33]. In addition, KLK6 has been identified as a marker of poor prognosis in colon and ovarian cancer patients [34, 35]. Natural and recombinant kallikrein inhibitors are cytotoxic to a wide range of human cancer cells *in vitro* [44, 45], but no data are available to demonstrate chemopreventive or chemotherapeutic efficacy *in vivo*. In this regard, protease inhibitors such as Bowman-Birk Inhibitor Complex (BBIC) have been demonstrated to inhibit carcinogenesis in a broad range of experimental models (reviewed in [46]), including the hamster cheek pouch model of oral neoplasia [47]. However, although data from early oral cancer chemoprevention trials with BBIC were promising [48, 49], a recent Phase IIb randomized trial of BBIC in patients with oral leukoplakia found no significant differences in response in patients treated with BBIC or placebo [50].

A major challenge to the prevention of oral cancer is the lack of validated biomarkers that can be used to monitor the progression of oral lesions from premalignant (*e.g*., leukoplakia) to invasive cancers. Because the 4-NQO/F344 rat oral carcinogenesis model recapitulates many of the histologic and molecular changes seen in human oral cancers, it provides a useful platform for the identification of biomarkers of oral cancer development and progression [12]. In the present study, twelve of the 20 genes demonstrating the greatest degree of down-regulation in OSCC induced by 4-NQO were keratins or keratin-associated proteins. Although the reduced expression of these genes most likely does not identify them as novel targets for oral cancer chemoprevention, these data do suggest that altered keratin profiles may provide a useful biomarker of oral malignancy.

While genes that demonstrate the greatest differential expression in oral cancers provide rational targets for the design of drugs for oral cancer chemoprevention, it is clear that oral carcinogenesis can also be inhibited by modulation of genes and pathways that do not demonstrate the very large fold-changes in expression seen with the lipocalins, chemokines, and KLK6. A prime example of such a target is PTGS2 (COX-2). We have previously reported (and have confirmed in the present studies; Table 6) that PTSG2 is upregulated by approximately 12 to 16-fold in OSCC induced by 4-NQO. Oral carcinogenesis in this model can be inhibited by non-toxic doses of either specific (celecoxib) or non-specific (piroxicam, naproxen, NO-naproxen) inhibitors of COX-2 [14].

Our methylation analyses demonstrate that PTGS2 is upregulated in oral cancers through an epigenetic mechanism involving hypomethylation of the PTGS2 proximal promoter; interestingly, significant hypomethylation was seen outside of the CpG island region of the promoter. Altered methylation of the PTGS2 promoter has been reported to regulate PTGS2 expression in a number of organ sites and in response to stimuli as varied as cigarette smoke and pharmacologic agents [51–53]. The site of altered methylation within the PTGS2 promoter may be tissue- or stimulus-specific, as altered methylation of both promoter and the first exon of PTGS2 have been reported [51–53].

Methylation analysis also demonstrated that one down-regulated tumor suppressor gene, APC2, is hypermethylated in OSCC induced by 4-NQO. By contrast, down-regulation of four other tumor suppressor genes (CCNA1, HRASLS, DDIT4L, WIF1) could not be attributed to promoter hypermethylation. It should be noted, however, that promoter hypermethylation cannot be definitively ruled out as a mechanism for the underexpression of these four genes, since areas of hypermethylation could be present outside of those that were analyzed. Supporting this possibility are our data with PTGS2, which demonstrated that promoter regions beyond CpG islands can be important targets of DNA methylation.

The central goal of the present studies was to identify molecular pathways that may serve as useful targets for cancer chemoprevention in the oral cavity. We propose that on the basis of their substantial overexpression in rat oral cancers, lipocalins and chemokines are potentially suitable molecular targets for oral cancer prevention. This idea is further supported by reports that (a) lipocalins such as LCN2 and several chemokines are significantly overexpressed in human oral cancers [17, 24, 25] and (b) these and related targets appear to be “druggable” [23, 28, 29]. Based on the overexpression of KLK6 in both rat OSCC and several types of human malignancies [30–33], a similar argument can be made in support of further oral cancer chemoprevention studies with serine protease inhibitors. However, enthusiasm for this approach may be reduced by the lack of significant chemopreventive activity of another protease inhibitor, BBIC, in a recently reported clinical trial of leukoplakia patients [50].

Confirming and extending our previous results [14], PTGS2 and several COX-related genes were significantly upregulated in rat oral cancers induced by 4-NQO; upregulation of PTGS2 is associated with hypomethylation of its proximal promoter region. Down-regulation of the tumor suppressor gene, APC2, also appears to be mediated by epigenetic mechanisms involving increased methylation of its first exon. Overexpression of pro-inflammatory chemokines, hypomethylation and overexpression of PTGS2, and hypermethylation and underexpression of APC2 may all be causally linked to the etiology of oral cancer in this model. Our studies provide important clues for further studies directed at the identification of molecular pathways that may serve as mechanistically-based targets for chemoprevention of oral carcinogenesis.

## Supporting Information

S1 Fig Differentially expressed genes in 11 pairs of OSCC *vs.* phenotypically normal tissue (N) (3538 probes; >2-fold differential expression; paired T-test p-value < 0.05). Expression of each gene is normalized to the 75th percentile intensity of each array and further normalized to the mean expression within each tissue pair. Red/Orange = up-regulated genes in tissue pair; Yellow = comparable gene expression in T and N; Blue = down-regulated genes in tissue pair.
(TIF)

S2 Fig qRT-PCR confirmation of microarray data demonstrating up-regulation of selected genes in OSCC. qRT-PCR and microarray analyses were performed using different sets of OSCC and normal tissues. Data are expressed as mean ± SD, n = 8; *p < 0.05 in OSCC versus normal tissue.
(TIF)

S3 Fig Identification of one CpG island (345 to 494 bp) in 600 bp 5’ flanking sequence of proximal promoter of rat PTGS2 gene. 4 primers (P1-P4) covering the CpG island and non-CpG island regions were designed for methylation assays. TSS, transcription start site; numbers in box represent the location of primers.
(TIF)

S1 File Tables A-C. **Table A.** Specific primers used for RT-PCR analysis. **Table B.** Specific primers used for methylation assay of proximal promoters (600 bp 5’flanking sequence) of the selected genes (first exon starts from the position of 601 bp of the sequence). **Table C.** Expression of the selected putative tumor suppressor genes in 4-NQO-induced rat oral cancer.
(DOCX)
