# Supplementary figures and images for: Overexpression of Lipocalins and Pro-Inflammatory Chemokines and Altered Methylation of PTGS2 and APC2 in Oral Squamous Cell Carcinomas Induced in Rats by 4-Nitroquinoline-1-Oxide
Source: PLoS One. 2015 Jan 30;10(1):e0116285. doi: 10.1371/journal.pone.0116285 (PMC4312057; doi:10.1371/journal.pone.0116285)

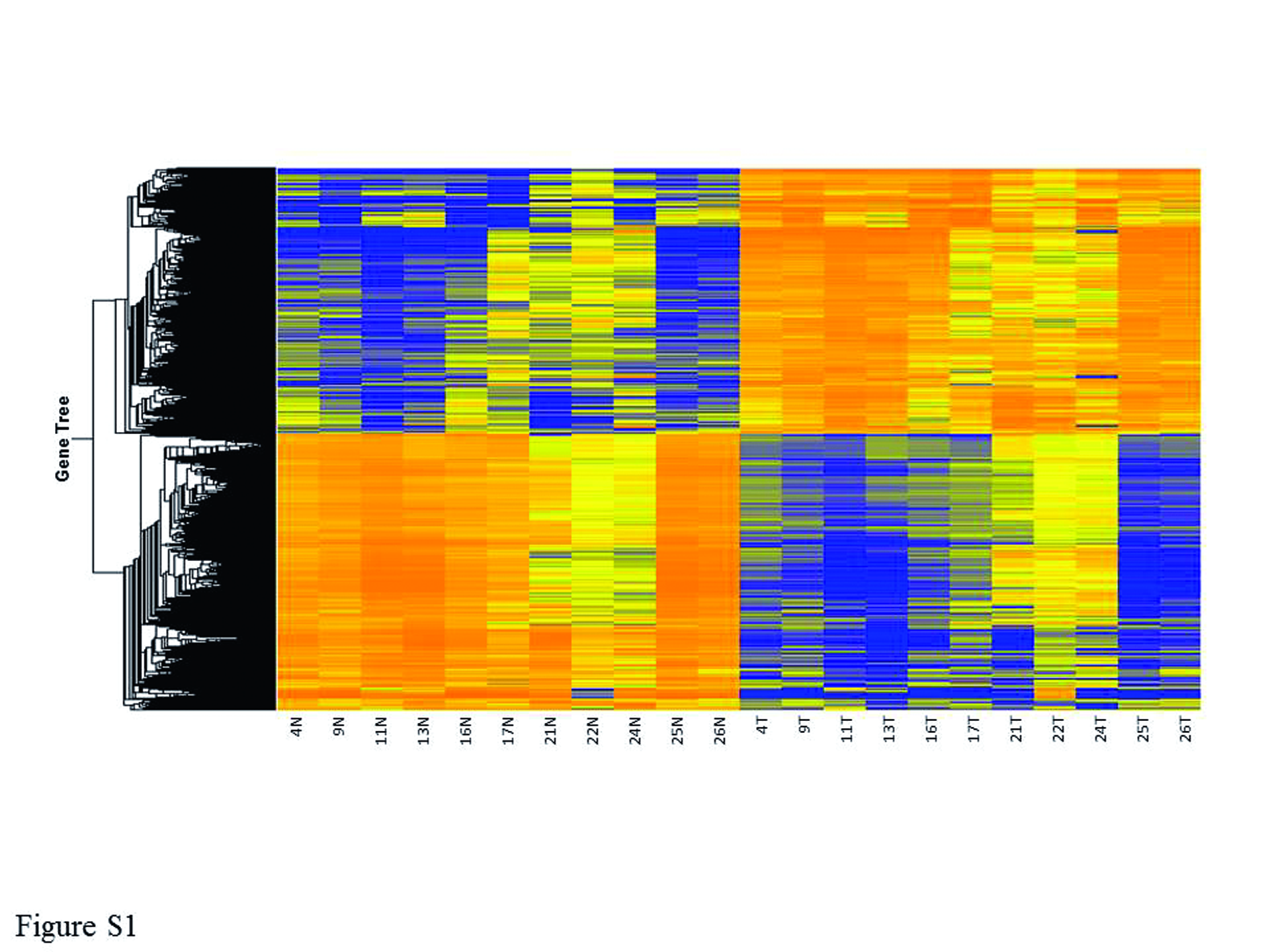

Supplement: S1 Fig — Expression of each gene is normalized to the 75th percentile intensity of each array and further normalized to the mean expression within each tissue pair. Red/Orange = up-regulated genes in tissue pair; Yellow = comparable gene expression in T and N; Blue = down-regulated genes in tissue pair. (TIF) [file pone.0116285.s001.tif]

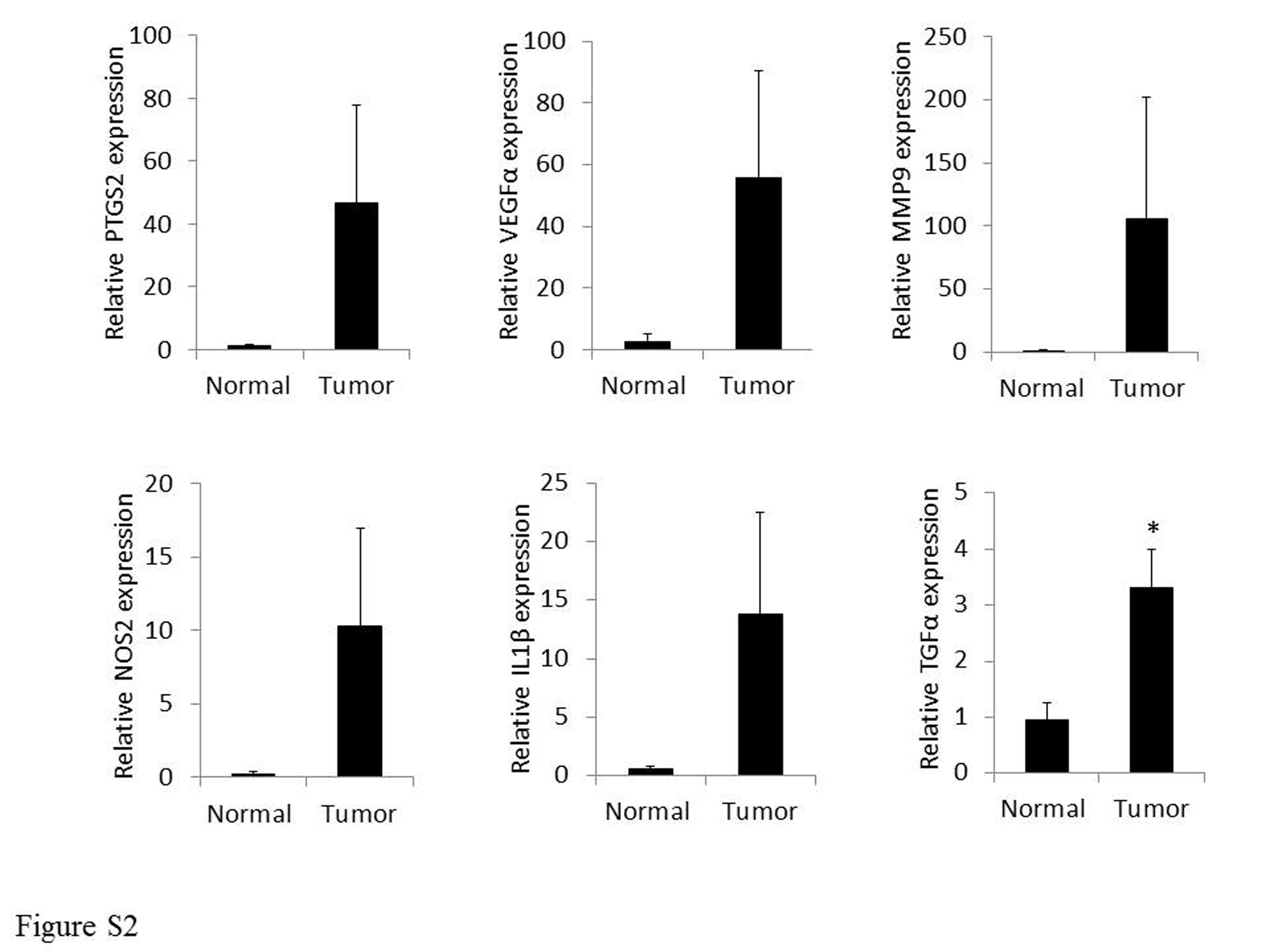

Supplement: S2 Fig — qRT-PCR and microarray analyses were performed using different sets of OSCC and normal tissues. Data are expressed as mean ± SD, n = 8; *p < 0.05 in OSCC versus normal tissue. (TIF) [file pone.0116285.s002.tif]

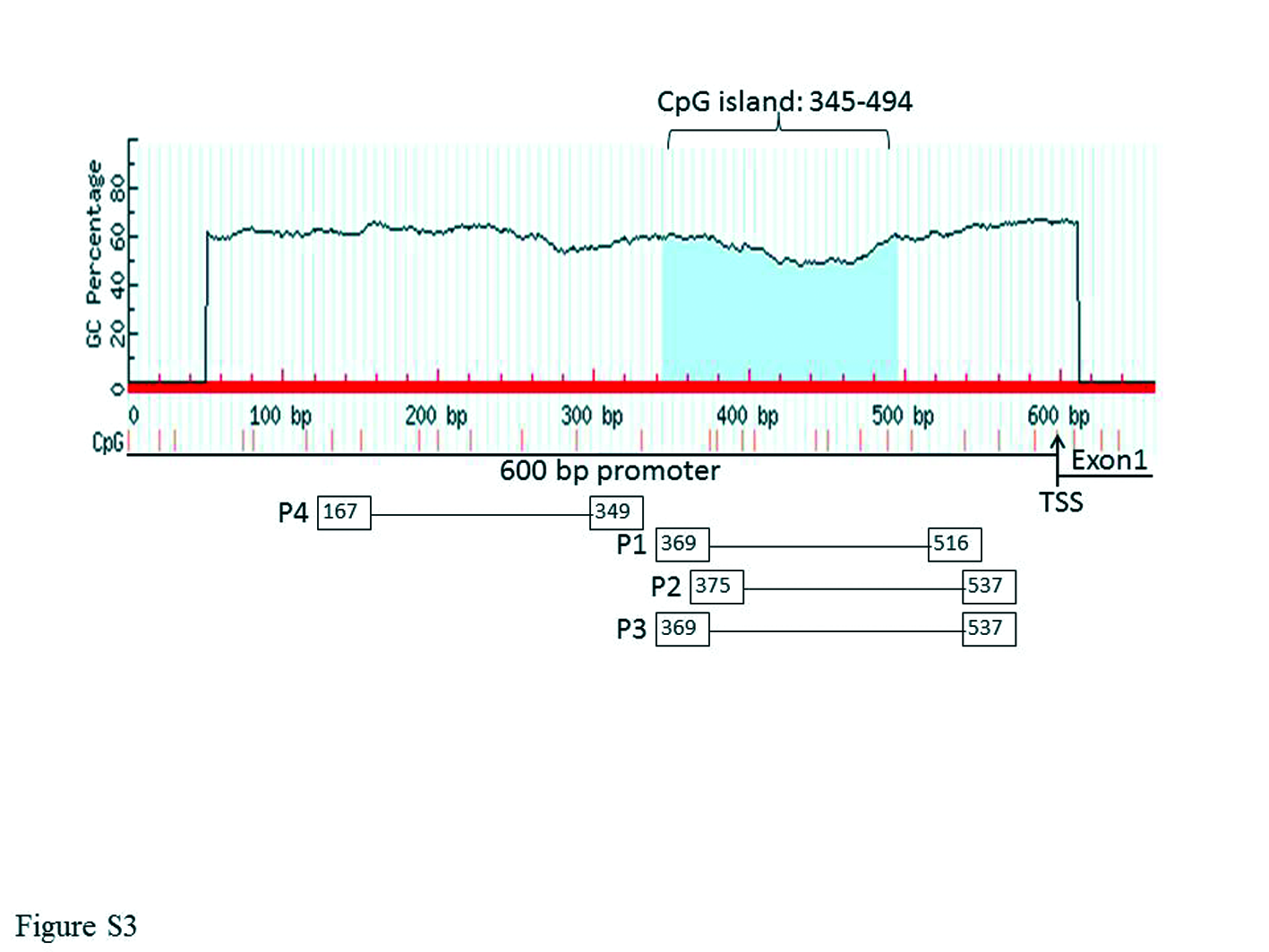

Supplement: S3 Fig — 4 primers (P1-P4) covering the CpG island and non-CpG island regions were designed for methylation assays. TSS, transcription start site; numbers in box represent the location of primers. (TIF) [file pone.0116285.s003.tif]
