# Supplementary material for: Overexpression of Lipocalins and Pro-Inflammatory Chemokines and Altered Methylation of PTGS2 and APC2 in Oral Squamous Cell Carcinomas Induced in Rats by 4-Nitroquinoline-1-Oxide
Source: PLoS One. 2015 Jan 30;10(1):e0116285. doi: 10.1371/journal.pone.0116285 (PMC4312057; doi:10.1371/journal.pone.0116285)
Supplement: S1 File — Table A. Specific primers used for RT-PCR analysis. Table B. Specific primers used for methylation assay of proximal promoters (600 bp 5’flanking sequence) of the selected genes (first exon starts from the position of 601 bp of the sequence). Table C. Expression of the selected putative tumor suppressor genes in 4-NQO-induced rat oral cancer. (DOCX) [file pone.0116285.s004.docx]

Table A. Specific primers used for RT-PCR analysis

Gene name Accession no. Primer sequence product size

PTGS2 NM_017232.3  F: TCAGAAGCGAGGACCTGGG

R: TACACCTCTCCACCGATGAC 147 bp

VEGFα NM_031836 F: CGGGCCTCTGAAACCATGAA

R: GCTTTCTGCTCCCCTTCTGT 122 bp

MMP-9 NM_031055 F: GATCCCCAGAGCGTTACTCG

R: GTTGTGGAAACTCACACGCC 132 bp

MMP-10 NM_133514.1 F: TCCCAGGAATTGAGCCACAAG

R: CAGCCAGCTGTTGCTCTTCAG 137 bp

MMP-12 NM_053963 F: ACGAGAGCGAATTTGCTGAA

R: GCCCAGTTACTTCTAGCCCA 150 bp

MMP-13 NM_133530.1 F: TCCATCCCGAGACCTCATGT

R: GCAGCACTGAGCCTTTTCAC 135 bp

TGFα NM_012671.2 F: GCCCTGGCTGTCCTCATTATC

R: ACTGTCTCAGAGTGGCAGCA 149 bp

IL-1β NM_031512.2 F: TGGCAACTGTCCCTGAACTC

R: CCCAAGTCAAGGGCTTGGAA 121bp

iNOS NM_012611.3 F: TCAGGCTTGGGTCTTGTTAGC

R: GAAGAGAAACTTCCAGGGGCA 110 bp

18S-rRNA NR_046237.1 F: CGAAAGCATTTGCCAAGAAT

R: AGTCGGCATCGTTTATGGTC 102 bp

Table B. Specific primers used for methylation assay of proximal promoters (600 bp 5’flanking sequence) of the selected genes (first exon starts from the position of 601 bp of the sequence)

Gene name CpG island location Primer location and sequence product size

PTGS2 345-494 167F: GCCAGTCTTGGAGCAGGCACAGC

349R: AGCTTTCCCCCATCTCCGCTTCC 183 bp

CCNA1 437-604 453F: AGGGCAGGCGTCCTGGAGAAGG

615R: CACGAACCCCTCCCGCTAACGTC 163 bp

HRASLS 462-605 454F: GGGTCTGGGGCGAGGTTCAAAGAC

650R: GACTCCATCCGCAGCACCCTGAG 196 bp

DDIT4L 395-529 351F: CTTGGCAGGAACTGAAAGGTGACCTGA

512R: GCCCACCAGCACAGGCTGCAC 162 bp

WIF1 191-310,325-604 Primers were purchased from Qiagen

Cat No. EPRN107710-1A

APC2 No CpG island 577F: CCCCAGGCACTGCAGGAGTTGAAA

760R: CCTCCATGCCGGTTCTCACCTTCA 184 bp

CpG island was identified by MethPrimer program.

Table C. Expression of the selected tumor suppressor genes in 4-NQO-induced rat oral cancer in comparison to the adjacent normal tissues identified by microarray analysis

| Gene Symbol | Primary Accession | Ratio (T/N) | P-value | Mean (N) | SD (N) | Mean (T) | SD (T) | Gene Name |
| --- | --- | --- | --- | --- | --- | --- | --- | --- |
| CCNA1 | NM_001011949 | 0.26 | 3.91E-04 | 1.43 | 0.45 | 0.37 | 0.40 | Cyclin A1 |
| HRASLS | NM_001105871 | 0.24 | 1.93E-04 | 1.47 | 0.43 | 0.36 | 0.37 | HRAS-like suppressor |
| DDIT4L | NM_080399 | 0.09 | 2.08E-04 | 1.63 | 0.43 | 0.15 | 0.38 | DNA-damage-inducible transcript 4-like |
| WIF1 | NM_053738 | 0.35 | 3.34E-05 | 1.41 | 0.32 | 0.50 | 0.28 | Wnt inhibitory factor 1 |
| APC2 | NM_001106769 | 0.48 | 7.73E-07 | 1.33 | 0.20 | 0.64 | 0.17 | adenomatosis polyposis coli 2 |

All values are derived from raw intensity values normalized to the 75th percentile of each array followed by the mean expression within each pair. P-values are derived from a paired Student T-test using GeneSpring software (n=11, p<0.01). Numbers were rounded to the nearest hundredth after calculations. T, tumor; N, Normal. These genes were selected based on literature and microarray for methylation analysis of their promoters.
